# Violaxanthin de-epoxidase disulphides and their role in activity and thermal stability

**DOI:** 10.1007/s11120-015-0118-9

**Published:** 2015-03-13

**Authors:** Erik Ingmar Hallin, Kuo Guo, Hans-Erik Åkerlund

**Affiliations:** Department of Biochemistry and Structural Biology, Lund University, P.O. Box 124, 221 00 Lund, Sweden

**Keywords:** Violaxanthin de-epoxidase, Cysteine, Mutation, Disulphide bond, Violaxanthin, Zeaxanthin

## Abstract

Violaxanthin de-epoxidase (VDE) catalyses the conversion of violaxanthin to zeaxanthin at the lumen side of the thylakoids during exposure to intense light. VDE consists of a cysteine-rich N-terminal domain, a lipocalin-like domain and a negatively charged C-terminal domain. That the cysteines are important for the activity of VDE is well known, but in what way is less understood. In this study, wild-type spinach VDE was expressed in *E. coli* as inclusion bodies, refolded and purified to give a highly active and homogenous preparation. The metal content (Fe, Cu, Ni, Mn, Co and Zn) was lower than 1 mol% excluding a metal-binding function of the cysteines. To investigate which of the 13 cysteines that could be important for the function of VDE, we constructed mutants where the cysteines were replaced by serines, one by one. For 12 out of 13 mutants the activity dropped by more than 99.9 %. A quantification of free cysteines showed that only the most N-terminal of these cysteines was in reduced form in the native VDE. A disulphide pattern in VDE of C9–C27, C14–C21, C33–C50, C37–C46, C65–C72 and C118–C284 was obtained after digestion of VDE with thermolysin followed by mass spectroscopy analysis of reduced versus non-reduced samples. The residual activity found for the mutants showed a variation that was consistent with the results obtained from mass spectroscopy. Reduction of the disulphides resulted in loss of a rigid structure and a decrease in thermal stability of 15 °C.

## Introduction

Plants and algae need light to drive the photosynthetic machinery. However, an excess of light will cause oxidation damage. To protect against the surplus of light, the energy is converted to heat, in a process called non-photochemical quenching (NPQ). One prerequisite for NPQ is the presence of zeaxanthin/antheraxanthin formed from violaxanthin in the xanthophyll cycle (Demmig-Adams 1990). This conversion is catalysed by violaxanthin de-epoxidase (VDE), located at the lumen side of the thylakoids. When exposed to intense light, the inside of the thylakoids will become acidic, which activates VDE and the conversion from violaxanthin to zeaxanthin. For a review on the xanthophyll cycle background see (Eskling et al. 1997). VDE consists of three domains, a cysteine-rich N-terminal domain with 11 of the total 13 cysteines, a lipocalin-like domain, predicted to bind violaxanthin (Saga et al. 2010) and a glutamate-rich C-terminal domain. The C-terminal is highly variable between different species and has been partially truncated without major loss of activity (Hieber et al. 2002). The three-dimensional structure of a dimer of the lipocalin domain has been determined (Arnoux et al. 2009) and binding of the substrates, violaxanthin and ascorbic acid, to this dimer has been suggested from in silico experiments (Saga et al. 2010). However, the N-terminally truncated protein used for structure determination had no enzymatic activity. Thus, the role of the cysteine-rich N-terminal remains to be established. The cysteines are conserved throughout all known VDE sequences with the exception of the first cysteine (Cys 7), which is not present in algae VDE. This wide conservation among different organisms is an indication of the cysteine’s importance for the function of VDE. An isolated *Arabidopsis thaliana* mutant (*npq*1, Niyogi et al. 1998), unable to convert violaxanthin to zeaxanthin, have exchanged one of these cysteines (Cys 72) for a tyrosine. The inhibition of VDE by dithiothreitol (DTT) suggests that disulphide bonds are necessary for the catalysis (Yamamoto and Kamite 1972). Cysteines in general are also known to coordinate metal ions, participate in catalysis, act as redox sensors or provide structural stability in the form of disulphides.

In this study, we have investigated the role of the cysteines in VDE by mutating all of the cysteines to serines, one by one, which resulted in the loss of activity of all cysteine mutants except for the first cysteine in the sequence (Cys 7). Metal analysis of purified active VDE did not reveal any metals that could potentially be bound to these cysteines. Quantification of free cysteines indicated only one free cysteine per VDE molecule, which suggests that the rest of the 12 cysteines form disulphides. The disulphide pattern was analysed with mass spectroscopy and revealed five out of six possible disulphide bonds. These bonds could give valuable information about the structure of the N-terminal domain. The thermal stability of VDE was reduced upon reduction with DTT, which indicates that one function of the disulphides is to maintain a rigid structure.

## Materials and methods

### Gene and original plasmid source

The original isolation of the spinach VDE gene sequence is described in (Emanuelsson et al. 2003). The mature native sequence of spinach VDE (VDALK…IRKLR) was inserted in pET22b+ (Novagen) between the restriction sites *Nde*1 and *Bam*H1 yielding a non-tagged protein with an initial methionine.

### Mutation protocol

The 13 cysteine mutants, where one cysteine per mutant have been exchanged to a serine, were constructed using the overlap extension polymerase chain reaction (Ho et al. 1989) with primers, shown in supplemental data, on pET22b+ containing the native sequence of spinach VDE. The amplicon of each mutant was inserted in pET22b+ between the restriction sites *Nde*1 and *Bam*H1.

### Protein isolation and purification

Overexpression of recombinant VDE was done in *Escherichia coli* BL21(DE3) grown in lysogeny broth at 37 °C, induced with isopropyl β-d-1-thiogalactopyranoside (1 mM) at OD_600_ ≈ 0.6 for 2 h at 37 °C. The cells were harvested by centrifugation, washed with Tris–HCl (100 mM, pH 8.0), NaCl (170 mM) and disrupted by French press in Tris–HCl (20 mM, pH 8.0), EDTA (10 mM), DTT (5 mM) and Triton X-100 (1 %, v/v). Inclusion bodies were isolated by centrifugation, washed three times with Tris–HCl (20 mM, pH 8.0), EDTA (10 mM), DTT (5 mM), Triton X-100 (1 %, v/v) and once without Triton X-100. The washed inclusion bodies were dissolved in urea (8 M), Tris–HCl (20 mM, pH 8.0), EDTA (10 mM), DTT (5 mM) and centrifuged to remove insoluble material. The refolding of the solubilised inclusion bodies were done by removing urea with a PD-10 gel filtration column, followed by incubation for 60 min and another PD-10 step to remove DTT. Reduced glutathione (2 mM) and oxidised glutathione (0.3 mM) were added and the mixture was incubated for 16 h at 22 °C. The refolded protein was purified by size-exclusion chromatography (HiPrep 26/60 Sephacryl S-200) in Tris–HCl (20 mM, pH 8.0), EDTA(10 mM), and NaCl (100 mM), collecting the monomeric VDE fraction and concentrating the protein using a 10 kDa cut-off centrifugation filter. The protein concentration was determined using the Bradford method (Bradford 1976) and the theoretical absorbance at 280 nm. The VDE activity was measured at 26 °C in citrate–phosphate buffer (50 mM citrate, 110 mM phosphate, pH 5.2), violaxanthin (0.33 µM, extracted from spinach leaves according to Thayer and Björkman 1990), monogalactosyldiacylglycerol (9 µM) and ascorbate (30 mM) by dual-wavelength measurements (502–540 nm), using a Shimadzu UV-3000 spectrophotometer according to (Yamamoto and Higashi 1978).

### Isolation and activity of cysteine mutants

Induced and washed *E. coli* BL21(DE3) cells (40 mg) with overexpressed native or mutated VDE were resuspended in 1.0 ml Tris–HCl (85 mM, pH 8.0), NaCl (85 mM), EDTA (0.85 mM), Triton X-100 (1 %, v/v) and lysozyme (0.5 g/l). After five freeze/thaw cycles, CaCl_2_ (10 mM) and DNase I (0.1 g/l) were added followed by incubation for 30 min, and centrifugation to collect the inclusion bodies. The pellet was washed two times with Tris–HCl (90 mM, pH 8.0), NaCl (90 mM), EDTA (0.9 mM), Triton X-100 (1 %, v/v) and once without Triton X-100. The inclusion bodies were dissolved with urea (8 M), Tris–HCl (60 mM, pH 8.0), NaCl (60 mM), EDTA (0.6 mM) and centrifuged to remove insoluble material. The supernatant was dialysed against 50 ml of Tris–HCl (100 mM, pH 8.0), NaCl (100 mM), EDTA (1 mM) for 16 h at 22 °C using 10–14 kDa dialysis tubing (Spectra/Por). The refolded protein was centrifugated at 100,000×*g* for 60 min to remove the aggregated protein.

The VDE activity was measured by mixing refolded VDE with citrate–phosphate buffer (50 mM citrate, 110 mM phosphate, pH 5.2), violaxanthin (8.6 µM, extracted from spinach leaves according to Thayer and Björkman 1990, monogalactosyldiacylglycerol (24 µM), ascorbate (30 mM) and incubated at 22 °C for 270 min. After the reaction, the pH was raised to seven with NaOH. The xanthophylls were extracted by addition of acetone (80 % final concentration) and centrifuged to remove insoluble material. The solvent of the supernatant was evaporated with a vacuum concentrator (Christ, RVC 2-18), to be replaced with ethyl acetate (100 %) and centrifuged to remove insoluble material, followed by another solvent exchange to methanol (100 %). The concentrations of violaxanthin, antheraxanthin and zeaxanthin were quantified by reversed-phase HPLC (Waters 600E, 996), as described in (Clausén et al. 2010).

### Quantification of free cysteines

The number of thiols in VDE was quantified using 5,5′-dithiobis-(2-nitrobenzoic acid) (DTNB), similar to (Sedlak and Lindsay 1968) in Tris–HCl (200 mM, pH 8.5), EDTA (1.0 mM), SDS (0.5 % w/v), DTNB (0.2 mM) and 2.9 µM wild-type VDE or C7S mutant, using a Shimadzu UV–Vis spectrophotometer (UV-2101PC) and l-cysteine as calibration standard.

### Mass spectroscopy

Enzymatic digestion of purified VDE (10 µg) was performed by incubation with thermolysin (1 µg, from Promega) in 60 µl Tris–HCl (50 mM, pH 8.0), CaCl_2_ (1 mM) at 80 °C for 30 min. For reduction of disulphides 1 µl of DTT (500 mM) was added to 10 µl of the peptide sample and incubated for 20 min at 22 °C. Acidification of both reduced and non-reduced samples were done by the addition of trifluoroacetic acid (TFA) to 0.5 % (v/v). The peptide samples (0.5 µl) were spotted on the MALDI-target plate together with 0.5 µl of the matrix (α-cyano-4-hydroxycinnamic acid (10 mg/ml) in 70 % acetonitrile, 29 % water, 1 % TFA). Alternatively, the non-reduced peptides (after addition of TFA) were separated by reverse-phase nano-LC using a 1100 Series Nanoflow LC system (Agilent Technologies, Waldbronn, Germany) with a precolumn (Zorbax 300 SB C18, 5 × 0.3 mm) and separation column (Zorbax 300 SB C18, 150 × 0.075 mm). Peptides were eluted with an acetonitrile gradient (1–90 % acetonitrile in water, with 0.1 % TFA) and collected on a stainless steel MALDI-target using an 1100 Series LC microcollection spotting system. For reduction of separated peptides, TCEP (0.3 µl, 10 mM) was added to the MALDI-target before addition of the matrix (0.2 µl, same concentrations as above). MS and MS/MS spectra were collected using a 4700 MALDI TOF/TOF mass spectrometer (Applied Biosystems, Framingham, CA, USA) in positive reflector mode.

### Metal analysis

The concentration of Fe, Cu, Ni, Mn, Co and Zn was measured by inductively coupled plasma mass spectrometry (ICP-MS) for purified VDE (14 µM), with added nitric acid (1 %), using an Aurora Elite (Bruker).

### Thermal stability measurement with dynamic light scattering

The thermal stability was measured for purified VDE (18 µM) in Tris–HCl (60 mM, pH 8.0), NaCl (100 mM) and EDTA (5.5 mM), using a Zetasizer Nano S (Malvern Instruments, Malvern, UK). The reduced VDE sample also contained DTT (10 mM). The z-average cumulant size (ISO 13321 1996) was recorded at 3 °C intervals from 15 to 70 °C with 60 s of equilibration time.

## Results

### Protein characteristics

The recombinant spinach VDE expressed in *E. coli* as inclusion bodies, refolded and purified as a monomeric protein was >98 % pure based on SDS-PAGE and Coomassie staining (not shown). The enzyme was highly active and showed a specific activity of 208 µkat/g protein (750 µmol/mg/h), which is similar to the value for native VDE purified from spinach (Arvidsson et al. 1996). The final yield of enzyme was 2 mg/l of growth medium. The recombinant protein did not show any obvious prosthetic group, since no absorbance could be detected in the visible range of the spectra even at concentrations as high as 10 mg/ml (not shown). Some prosthetic groups may involve metal ions bound to cysteine ligands without showing any absorbance. To check this possibility, metals that potentially could be bound to cysteines were analysed in the purified recombinant and active VDE. The metals Fe, Cu, Ni, Mn, Co and Zn were all found in amounts less than 1 % of VDE on a molar basis and are therefore neither not likely to be bound to the enzyme nor required for the enzymatic activity.

### Violaxanthin de-epoxidase activity of the cysteine mutants

**Fig. 1:**
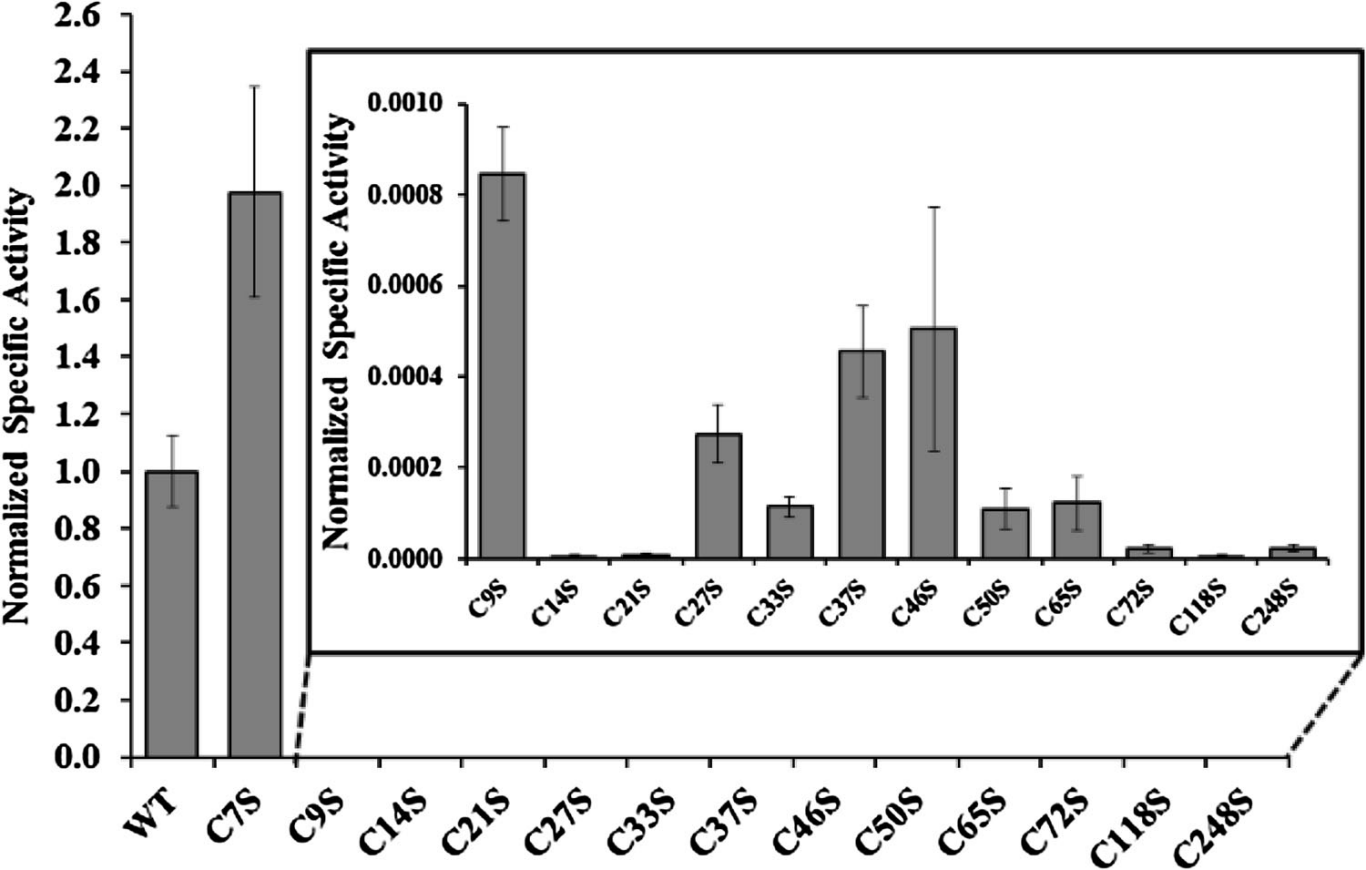
The specific activity of the cysteine mutants, normalised to the specific activity of native spinach VDE (WT). A magnification of the mutants with low activity is shown in a window to visualise the difference in remaining activity

The mutation of a cysteine to a serine resulted in an activity loss (Fig. 1) of more than 99.9 % for all cysteine mutants except for C7S. Thus all cysteines, except C7, are important for the enzyme function, and are consistent with the conservation of these cysteines throughout evolution. The low activity of C72S mutant, corresponding to the cysteine mutated in *npq*1, is coherent with the incapability of *npq*1 to accumulate zeaxanthin (Niyogi et al. 1998). Even though most of the mutants lost almost all activity, all of them still were catalytically active and produced both antheraxanthin and zeaxanthin. As the degree of conversion of violaxanthin was very low under the conditions used here, the dominating product was antheraxanthin, but zeaxanthin could also be clearly detected (not shown). A very low activity from a potentially inactive mutant could be possible due to translational misreading errors, which has been estimated to occur in the range of 10^−3^–10^−4^ per codon (Kramer and Farabaugh 2007). Thus, a low amount of active VDE with the wild-type sequence could be present while most of the enzyme molecules were completely inactive, rather than that each enzyme molecule shows a low activity. However, the activities showed large differences depending on which cysteine was mutated, with less than 0.0005 % activity remaining for C14S and more than 100-fold higher activity remaining for the C9S mutant. The C7S mutant was completely different. It was not inhibited at all but showed even higher specific activity than the wild-type VDE. Thus, this cysteine is not important for the enzymatic function, under the assay conditions tested, and can be replaced by a serine. This could explain why Cys 7 is not conserved in algae VDE.

A basic question is if the cysteines in VDE are in the oxidised form as disulphides or in the reduced form as free cysteine groups. The quantification of free cysteines in the recombinant wild-type VDE gave a molar ratio of 0.87 ± 0.24 thiols per VDE molecule which indicates that wild-type VDE has only one free cysteine. In contrast, the C7S mutant gave a molar ratio of 0.28 ± 0.10 thiols per VDE. This value is closer to zero than two and indicates that Cys 7 is the free and reduced cysteine in wild-type VDE. All the other cysteines should be in the form of disulphides, but which cysteines are coupled to each other?

### Disulphide pattern

Thermolytic digestion of purified recombinant VDE without reduction of the cysteines will maintain the native disulphide bonds. To identify peptides containing a disulphide bond, reduced and non-reduced samples were compared. A mass peak, only found in the non-reduced sample and not in the reduced sample is an indication of a peptide containing a disulphide bond. If the disulphide bond was an internal linkage within the same peptide, the reduction will cause a mass shift of +2 Da. A disulphide bond between two peptides will, upon reduction, show two new mass peaks in the reduced sample. The mass of the two linked peptides will hint possible disulphide connections by combining the mass of other cysteine-containing peptides. Peptides containing a disulphide bond were confirmed by MS/MS, when at least three well-defined peaks were found supporting the prediction.

**Fig. 2:**
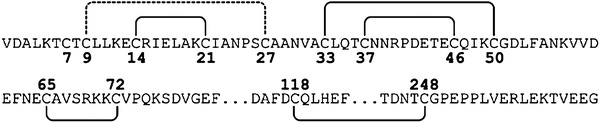
The disulphide bond pattern of spinach VDE. The *solid lines* represent disulphide bonds confirmed with mass spectroscopy data. The *dashed line* is the disulphide bond predicted by thiol quantification

By analysing the mass of peptides containing a disulphide bond, five out of six possible disulphide bonds could be identified (Table 1 and Fig. 2). No peptides with alternative disulphide connections were found. One of the disulphide bonds found, connects Cys 118 and Cys 248. This pair is located inside the lipocalin domain and the result is in agreement with the X-ray structure of the lipocalin domain of the recombinant VDE of *Arabidopsis thaliana* (Arnoux et al. 2009). No peptide representing Cys 7, Cys 9 or Cys 27 could be positively identified. If these cysteines form disulphides or not could therefore not be confirmed with mass spectroscopy. However, the results presented in the last section showed that it was only the Cys 7 that was in the reduced form, and all the other cysteines would be engaged in disulphide linkages. This suggests indirectly that the two remaining cysteines, Cys 9 and Cys 27, form one disulphide linkage with each other (dotted line in Fig. 2). As one possible role for the disulphides would be to stabilise the structure of VDE, we studied the heat denaturation of the non-reduced and reduced protein by dynamic light scattering.

**Table 1:** List of peptides containing a disulphide bond found using mass spectroscopy and confirmed with MS/MS

Disulphide bond	Observed mass (Da)	Theoretical mass (Da)	Observed mass after reduction (Da)	Theoretical mass after reduction (Da)	Sequence
14–21	1303.67	1303.69	1305.69	1305.70	LKE**C**RIELAK**C**
14–21	1416.76	1416.77	1418.79	1418.79	LLKE**C**RIELAK**C**
14–21	1434.77	1434.78	–	–	LLKE**C**RIE+AK**C**I
37–46	1648.67	1648.67	1650.67	1650.68	LQT**C**NNRPDETE**C**Q
118–248	1808.76	1808.76	–	–	FD**C**Q+IRTDNT**C**GPEPP
118–248	2521.09	2521.08	–	–	FDAFD**C**QLHE+IRTDNT**C**GPEPP
118–248	2567.14	2567.12	–	–	LNPTFDAFD**C**Q+IRTDNT**C**GPEPP
33–37–46–50	2696.17	2696.16	2700.19	2700.19	VA**C**LQT**C**NNRPDETE**C**QIK**C**GDLF
118–248	2714.21	2714.19	–	–	LNPTFDAFD**C**Q+FIRTDNT**C**GPEPP
37–46–50	2738.25	2738.23	2740.25	2740.25	LQT**C**NNRPDETE**C**QIK**C**GDLFANK
118–248	3018.47	3018.37	–	–	IRTDNT**C**GPEPPLVER+FDAFD**C**QLHE
118–248	3165.56	3165.44	–	–	FIRTDNT**C**GPEPPLVER+FDAFD**C**QLHE
65–72	3403.77	3403.63	3405.65	3405.65	VVDEFNE**C**AVSRKK**C**VPQKSDVGEFPVPDPS
65–72	3930.10	3929.98	3932.04	3931.99	VVDEFNE**C**AVSRKK**C**VPQKSDVGEFPVPDPSVLVKS

The mass of peptides with intramolecular disulphides after reduction are also shown. Intramolecular disulphides are shifted +2 Da after reduction, while reduction of intermolecular disulphides splits the linked peptides into two

### Thermal stability of reduced and non-reduced VDE

**Fig. 3:**
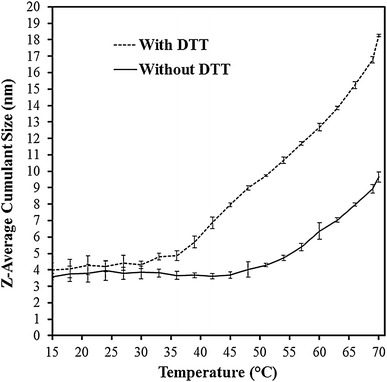
Temperature denaturation of spinach VDE in reduced (*dashed line*) and in non-reduced (*solid line*) form. VDE, reduced with DTT appears larger than non-reduced VDE and shows a decrease in melting temperature of 15 °C

The apparent melting temperature for the non-reduced VDE was found at around 48 °C (Fig. 3). After reduction with DTT, the melting temperature was lowered by 15 °C, to around 33 °C. This strong shift in melting temperature shows that the disulphides are indeed important to maintain the structure of VDE. The increased radius of reduced VDE also shows that the disulphides keep flexible domains in position. Size-exclusion chromatography of reduced and non-reduced VDE gives similar results, where reduced VDE elutes before non-reduced VDE (data not shown).

## Discussion

The results show that all cysteines in VDE, except the first one, are important for the enzymatic activity, form disulphides and do not seem to be involved in metal binding. Five out of six possible disulphide bonds were found using mass spectroscopy. A sixth disulphide bond is predicted to connect Cys 9 and Cys 27 since the quantification of free cysteines suggests only one free cysteine per VDE molecule for the native sequence and none for the C7S mutant, which means that Cys 7 should be the free, unbound cysteine. There is also the possibility that Cys 9 and Cys 27 have been chemically modified, therefore not reacting as thiols.

The disulphide pattern, obtained by mass spectroscopy, is not similar to the disulphide bonds suggested by disulphide prediction tools. DISULFIND (Ceroni et al. 2006) predicts no disulphides that match the disulphide pattern from our mass spectroscopy data. DiANNA (Ferrè and Clote 2005) gives only one disulphide bond with a probability score above 0.9 and agrees with the disulphide pattern in Fig. 2, which is the Cys 9–Cys 27 predicted disulphide. The weighted matching also predicts the Cys 14–Cys 21 disulphide, which our experimental data can support. The disulphide between Cys 118 and Cys 248 located inside the lipocalin domain, confirmed by the structure of VDE-lipocalin from *A. thaliana*, could not be predicted by either DISULFIND or DiANNA. That these prediction tools cannot match the correct disulphides could be an effect of the high number of cysteines, which the tools can not handle or that this disulphide pattern are very different from the ones used as basis for comparison in the prediction programs.

When comparing the disulphide pattern with the activity of the cysteine mutants, the activity for one disulphide-forming cysteine mutant was often similar to the activity of the mutant with a mutation of the corresponding disulphide-forming cysteine. If the loss of activity was caused only by the loss of a disulphide bond, the mutation of any of the two disulphide-forming cysteines would result in the same amount of activity loss. Therefore, the similar activity loss from a mutation of a cysteine, forming a disulphide bond, and a mutation of the other cysteine in this disulphide bond is an indication that it is the loss of the disulphide bond that causes the loss of activity.

The disulphide pattern obtained here is also expected to be present inside the plant cells in vivo despite that the inside of living cells generally has reducing conditions. The arguments for this is that the in vitro form of VDE needs all six disulphides to show full enzyme activity, and that VDE whether studied in vitro or within the leaf is inhibited by DTT. One possibility is that in the thylakoid lumen in the dark, the cysteines in VDE are in the reduced form. Upon illumination of leaves, photosynthesis starts to produce oxygen and makes the lumen more oxidising, which would allow disulphides to be formed. However, artificial acidification of whole leaves without illumination induces conversion of violaxanthin to zeaxanthin (Clausén et al. 2010). Thus, VDE can be active in the leaves in the dark and the disulphides should therefore be present also in the dark. The question then still remains how the disulphides in VDE are originally formed. The condition inside the thylakoid lumen may be less reducing than the cell as a whole to allow the formation of all the disulphide bonds required for the activity of VDE. That the increased conversion of violaxanthin to zeaxanthin caused by various stress treatment (Fernández-Marín et al. 2011) could be an effect of stronger oxidative conditions, which would favour the formation of disulphide bonds and an activation of VDE. The reactivation of reduced VDE is possible in vitro after the removal of reducing agent, as demonstrated by the procedure used in this work to make active VDE, showing that the correct disulphide bonds form spontaneously. In vivo this process could, however, also be catalysed by other proteins. Thioredoxin have been shown in vitro to interact and inactivate VDE (Hall et al. 2010). However, thioredoxin has not been found in the thylakoid lumen, but the lumen thiol oxidoreductase (LTO1/AtVKOR) may possibly fulfil this function. LTO1/AtVKOR is located in the thylakoid membrane with a thioredoxin-like domain oriented to the thylakoid lumen (Karamoko et al. 2011). Recent results from studies on an lto1-2 mutant, lacking functional LTO1 (Lu et al. 2013), and a yeast two-hybrid assay (Lu et al. 2014) show that both functional and physical interaction between LTO1 and VDE is possible.

The reaction catalysed by VDE is unusual, but the enzyme vitamin K epoxide reductase (VKOR) also catalyses de-epoxidation. In this case, vitamin K 2,3-epoxide is converted to vitamin K, in the blood coagulation process. This enzyme is suggested to utilise a disulphide bond that first is reduced, and then oxidised, to remove the epoxide and form a double bond (Silverman 1981; Davis et al. 2007). The possibility that a disulphide in VDE may be involved directly in the enzymatic reaction is therefore tempting. Considering that the conversion of violaxanthin to antheraxanthin is a de-epoxidation reaction to form a double bond and that VDE has disulphides that are important for this enzymatic reaction, a mechanism similar to the one in VKOR might be possible. The initial reduction of the disulphide in this mechanism might then be caused by ascorbate. However, more experimental evidences are needed to establish such a mechanism. An alternative to this mechanism is a concerted reaction without the involvement of disulphides, and that the disulphides only maintain a rigid structure of VDE. The loss of a more rigid structure is what is shown in Fig. 3, where reduced VDE appears to be larger than non-reduced VDE. A less rigid structure could be a signal for degradation of VDE, as for the case of PsbO, which after reduction of a disulphide bond becomes sensitive to proteolysis (Kieselbach 2013). This rigid structure could also be important for the protein stability in an environment that can undergo large shifts in pH, such as the thylakoid lumen, where the pH can vary from 5.7 to 7.5 depending on light conditions (Takizawa et al. 2007).

In conclusion, we have mutated the cysteines of spinach VDE one by one and observed major activity loss for 12 out of 13 mutants. Using mass spectroscopy five out of six possible disulphide bonds could be identified, while the sixth disulphide was indirectly identified. Reduction of these disulphides opens up the structure of VDE and decreases the thermal stability by 15 °C.
